# Knowledge, attitude, and practices toward sun exposure and use of sun protection among non-medical, female, university students in Saudi Arabia: A cross-sectional study^☆^

**DOI:** 10.1016/j.ijwd.2018.11.005

**Published:** 2019-01-23

**Authors:** Reema Ruddah Almuqati, Ali Saeed Alamri, Nawal Ruddah Almuqati

**Affiliations:** a Dermatology Department, Dr. Soliman Fakeeh Hospital, Jeddah, Saudi Arabia; b Department of Public Health, Ministry of Health, Jeddah, Saudi Arabia; c Dermatology Department, Munich University Hospital, Munich, Germany; d Security Forces Hospital, Riyadh, Saudi Arabia

**Keywords:** Sun exposure, sun protection, sunscreen

## Abstract

Excessive unprotected sun exposure is a significant risk factor for skin damage and skin cancers. In recent decades, the incidence of skin cancer has increased dramatically worldwide, reaching epidemic proportions. Skin cancer is the most common type of cancer worldwide, and the ninth most common malignancy in Saudi Arabia. Sun protection is a key primary preventive strategy against skin cancer and skin damage induced by sun exposure.

Herein, a cross-sectional study was made to evaluate the knowledge, attitude, and practices toward sun exposure, and the use of sun protection among non-medical female students on the Sulaymaniyah campus of the King Abdul-Aziz University. Also, we identified the reasons that prevented the students from using sun protection measures. A special-designed, self-administered questionnaire was applied on a sample size of 501 students. We found that most students were aware of the risks of unprotected sun exposure. Nevertheless, a view of sunscreen as harmful was reported (34.1%). Seeking shade and wearing protective clothing were the most used sun protection methods (58.1% and 43.1%, respectively). Sunscreen users made up only one third of our sample (23.6%). However, the majority of students (64.9%) did not know about the sun protection factor of sunscreen products. Discomfort felt on the skin was the most commonly reported reason for avoiding the use of sunscreen (40.7%). When comparing our study with Western studies, we found a high level of awareness among our sample of students. Significant differences in the attitude toward the application of suntan and sun protection products might be due to differences in cultural background. Our results spotted the need for future health education programs for our society that focus on the significant importance of sunscreen, as well as the correct methods of application.

## Introduction

Excessive unprotected sun exposure has been shown to cause skin damage as well as many skin diseases (McKenzie et al., 2009). As a result of short-term exposure to ultraviolet (UV) radiation, human skin could suffer from acute damage, including burning and tanning. Furthermore, long-term UV exposure may result in chronic skin diseases, such as hyperpigmentation (e.g., solar lentigines, ephelides, and melasma), skin aging (e.g., telangiectasias and elastosis), and skin cancer (Plensdorf and Martinez, 2009, Polefka et al., 2012).

Skin cancer is the most common type of cancer worldwide, and the ninth most common malignancy in Saudi Arabia (Al-Eid and Manalo, 2010). In recent decades, the incidence of skin cancer has increased dramatically worldwide, and reached epidemic proportions (Cercato et al., 2015, Diepgen and Mahler, 2002). Since 1950, the rate of skin cancer has doubled in fair-skinned populations, but the rate has remained relatively low for darker skin groups (Fund WCR, 2007).

There were fewer cases of skin cancer among individuals with darker skin due to increased epidermal melanin, which provides greater photoprotection. The higher level of epidermal melanin filters twice as much UV radiation in comparison with the lower levels of epidermal melanin of Caucasians (Brenner and Hearing, 2008). Of note, the lower incidence of skin cancer among patients with a darker skin tone does not signify full immunity. These individuals are still susceptible to the development of skin cancer, and are in fact more likely to present with late-stage cancers (Bryant et al., 2015).

Sun protection is a key primary preventive strategy against skin damage induced by sun exposure. Avoiding sun exposure between 10:00 a.m. and 2:00 p.m., seeking shade, using broad-spectrum sunscreen products, and wearing wide-brim hats, protective clothing, and sunglasses are the main recommendations for efficient sun protection (Cheng et al., 2010).

Many sun protection programs have been implemented in numerous western nations to increase public awareness toward sun-exposure risk, and encourage the use of sun protection measures. An increase in the awareness among populations has been observed. Nevertheless, compliance with regard to sun protection remains insufficient (Jones et al., 2007, Saridi et al., 2016). In Saudi Arabia, there were few studies on this matter. Not much is known about the awareness of the Saudi population about the use of sun protection measures. Information from this study may be helpful in designing effective interventions.

Jeddah, a coastal city in Saudi Arabia with a population > 3 million people, has a hot desert climate and clear skies most of the year (Weather Spark, 2016).

Our survey aimed to investigate the knowledge, attitudes, and behaviors of non-medical female students on the Sulaymaniyah campus of the King Abdul-Aziz University (KAU) in Jeddah, Saudi Arabia, with regard to sun exposure and protection. We chose students to be investigated, because they were in the age range when sun prevention behaviors have been proven to prevent skin aging and cancer.

## Methods and materials

A cross-sectional survey was conducted of female students on the Sulaymaniyah campus of KAU in Jeddah, Saudi Arabia. According to the Dean of Admissions and Registration, the total population of the Sulaymaniyah campus is 10,821 female students over eight faculties.

Participants were selected in two stages. The first stage was a selection of three of the eight faculties by random sampling, which resulted in the selection of the Faculties of Arts and Humanities, Sciences, and Communication and Media. The second stage involved a stratified random sample from each college. Only the second, third, and fourth grades were considered, because the first grade is a preparatory year and almost not present on this campus. The final sample size was 501 students from three colleges with proportional allocation.

**The d**ata were collected between October and November 2016. All participants were informed of the demands of the study, and those who agreed to participate were enrolled. Verbal consent was obtained. Participants who refused to participate or failed to complete the questionnaires were excluded from the study.

The questionnaire used for the data collection was specifically designed and tested on a pilot group of 15 students to calculate the approximate completion time, verify the range of questions asked by students, and improve the questionnaire accordingly. These questionnaires were excluded from the concluding analysis. Also, the questionnaire reliability was tested using Cronbach's Alpha, with a 0.66 result. The validity was tested using the content validity index, with a score of 0.71. The final self-administered questionnaire included 36 questions, and required approximately 6 minutes to complete. The final questionnaire was approved by the ethics committee of KAU.

The questionnaire consisted of five parts. The first part included social demographic data, such as age, faculty, specialty, academic year, and skin type. The second part included 15 questions about the participant’s knowledge of sunscreen products, as well as the harmful effects of the sun on the skin. The third part included four questions about the participant’s attitude toward measures of sun protection, and the fourth part 14 questions about practices of sun protection. The fifth part included two questions about reasons that deter from using measures of sun protection.

## Statistical analysis

The Statistical Package for the Social Sciences Program, version 22, was used for the analysis of the data (SPSS Inc., Chicago, IL). Descriptive statistics in the form of frequency and percentage were used for qualitative data, and the mean and standard deviation for quantitative data. The X^2^ test was used for comparisons between knowledge and practices among nonmedical female students. Statistical significance was defined as a *p* < .05.

## Results

In total, 501 students completed the survey. There was a diverse range of age among the students, with a mean age of 21.229 years (range: 19-29 years; standard deviation: 1.243). The majority of the students (80%) had Fitzpatrick skin type V. The breakdown of students by academic year included 28.1% in the second year, 29.1% in the third year, 29.9% in the fourth year, 10.8% in the fifth year, and 2% in other academic years (Table 1).

**Table 1:** Demographic characteristics of the students

Variable (characteristics)	n (%)
Academic year	
2^nd^	141 (28.1)
3^rd^	146 (29.1)
4^th^	150 (29.9)
5^th^	54 (10.8)
Other	10 (2)

Age	
The mean age (year) + standard deviation	21.229 + 1.24
19	1 (0.2)
20	173 (34.5)
21	146 (29.1)
22	5 (21)
23	56 (11.2)
24	12 (2.4)
25	6 (1.2)
26	1 (.2)
29	1 (.2)

Skin type	
I	0
II	2 (4)
III	5 (1)
IV	89 (17.8)
V	401 (80)
VI	4 (0.8)

## Sun exposure and sunscreen knowledge

Most students (94.8%) agreed that sun exposure could cause sunburn, but only 78.8% were aware of the relationship between sun exposure and skin cancer. Comprehension of the association between sun exposure and skin aging was reported by 71.3% of students, and 79.2% showed an understanding of the association between sun exposure and skin hyperpigmentation. The majority of students (86.8%) were aware of the harmful consequences of tanning beds, and 83% of students knew that the period of exposure to sunlight that causes the most harm is between 10:00 a.m. and 2:00 p.m. The association between sun exposure and age was misunderstood by 54.7% of students, who were unaware that sun exposure is more harmful during childhood compared with adulthood.

Most students (93.6%) agreed that men must also be protected against sun exposure. Seventy-two percent of students agreed that sunscreen products are protective, but 34.1% agreed that sunscreen products are harmful to the skin in various ways.

The association of vitamin D level and sun exposure was familiar to 70.9% of students, who agreed that sunscreen products are not harmful to the vitamin D levels in the body. Approximately two thirds of students (67.7%) believed that sunscreen products are not necessary on cloudy days. Seventy-three percent of students agreed that a face cover (i.e., hijab) is not sufficient to replace sunscreen products. The percentage of students who were knowledgeable about the sun protection factor (SPF) and protection grade of ultraviolet A (PA) values of sunscreen products were 35.1% and 22.2%, respectively.

## Attitudes and practices toward sun protection measures

Seventy-four percent of students indicated that they find protecting their skin from the sun difficult, and the majority of students (83.6%) were worried about damage from sun exposure. Seventy-four percent of students were convinced that the harmful consequences of sun exposure can be avoided by personal protection. The majority of students (66.7%) did not believe that sun tans make them more attractive. Most students (82.6%) reported that they never have had a sun tan.

Many students declared that they were protecting themselves on the sunny campus by seeking shade (58.1%), wearing long-sleeve clothing, or an Abaya (long-sleeved robe that covers the entire body except the head; 43.1%), and wearing sunglasses (33.9%). Twenty-three percent reported that they were using sunscreen products, but 35.1% admitted that they had never used sunscreen products. Nineteen percent reported that they avoided sun exposure between 10:00 a.m. and 2:00 p.m. The least commonly used protective measure was wearing a hat (3.6%).

Sunscreen product users chose their product according to information from a variety of sources, including advice from a pharmacist (33.5%), SPF and PA values (32.6%), suggestions from a dermatologist (25.6%), and information from the media (8.2%). The most commonly used SPF value among sunscreen product users was 50 (35.1%), but 17.4% of students used sunscreen products with an SPF > 70. Just short of a third of students (30.2%) did not know the SPF value of their sunscreen product.

The majority of sunscreen product users (84.5%) responded that they applied their product 15 to 20 minutes before sun exposure. With regard to reapplication, 63.1% of students reported that they never reapply sunscreen, and only 3.7% admitted to regularly reapplying sunscreen products. Among the students who reapply sunscreen, 21% reapply every 4 hours, 9.1% every 3 hours, and 6.7% every 2 hours. Students applied sunscreen products mostly on their face, neck, and hands (39.3%), and 25.6% applied sunscreen products on their face alone, and 25.3% only on their faces and hands.

**Fig. 1:**
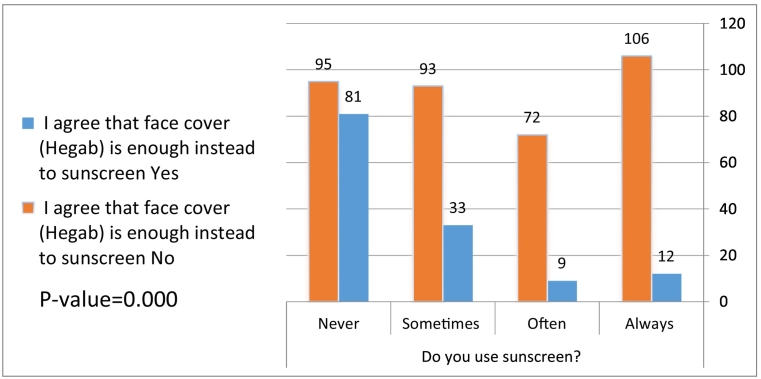
Correlation between face cover (hijab) and use of sunscreen products.

**Fig. 2:**
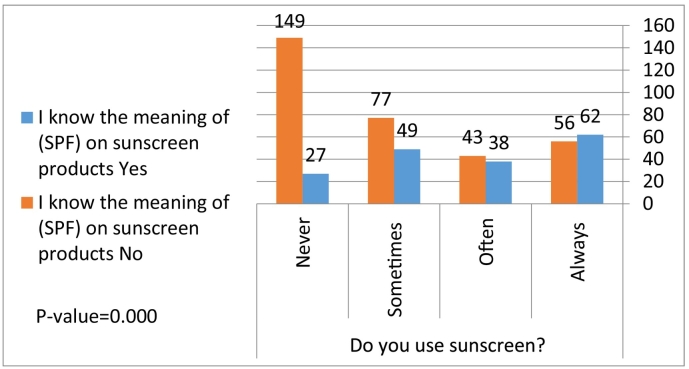
Correlation between knowledge of sun protection value value and use of sunscreen products.

When we compared students’ knowledge and behaviors with regard to sunscreen product use, there was a statistically significant relationship between the use of sunscreen products and the belief of sufficient protection of face cover (hijab) instead of sunscreen (*p* = .000; Fig. 1). In addition, there was a statistical relationship between the use of sunscreen products and the knowledge of the meaning of the SPF value (*p* = .000; Fig. 2).

## Reasons that prevent students from using photo protective measures

Among students who did not engage in protective measures, 70.7% reported that these measures caused them to feel uncomfortable, 16.2% were not convinced of the benefits of sun protection, and 13.2% were not aware of the harmful effects of sunlight. The most commonly reported reason for not using sunscreen was the feeling of discomfort of using sunscreen products on the skin (40.7%). Other reasons consisted of lack of time, lack of sunscreen awareness, high cost, and incompatibility with make-up products, which accounted for 28.9%, 15.7%, 7.5%, and 7.2%, respectively.

## Discussion

In Saudi Arabia, skin cancer ranks ninth among the cancer types for both men and women, with 319 cases accounting for 3.2% of the total amount of cancer diagnoses in 2010 (52.7% for men, 47.3% for women, male: female ratio = 111:100; Al-Eid and Manalo, 2010).

The rate of skin cancer among the Saudi population is low due to their darker skin type (Mufti, 2012). Another impact factor that has been identified is that the Saudi community is an Islamic conservative society, and has a cultural norm of full-body coverage of clothing, particularly for women. The majority of women cover their faces as well, which may explain the male predominance with the disease. However, the sun protection viability of these clothes is not well known. In addition, because Saudi Arabia has a hot desert climate, people are more inclined to participate in indoor activities.

There is very limited information on the knowledge and behavior of the Saudi people with respect to sun exposure and protection. We discovered only two studies conducted in Saudi Arabia that focused on this topic (Al Ghamdi et al., 2016, Al Robaee, 2010).

The results of our study indicated that female students were knowledgeable about the harmful consequences of sun exposure, including sunburn, hyperpigmentation, aging, and skin cancer. Their levels of awareness were higher than those in previous studies conducted in Saudi Arabia. For example, in the first study by Al Robaee (2010), only 56% of participants reported awareness of the relationship between sunburn and skin cancer, and in the second study by Al Ghamdi et al. (2016), only 55.3% of participants were aware of the relationship.

In our study, there was a significant increase in awareness of the risks of skin cancer, as reported by 78% of our participants. These differences are most likely due to the different demographic characteristics of the study population. In the current study, we focused on women within a specific age group (i.e., university students), because university students are at an age when sun prevention behaviors have proven to prevent skin aging and cancer. In addition, most students were concerned about skin damage, especially those who were exposed to sunlight at peak hours of sunshine from 10:00 a.m. to 2:00 p.m.

In comparison with western studies, we found higher levels of awareness among university students from western communities, especially on the association between skin cancer and sun exposure. A study from the United States by Spradlin et al. (2010) evaluated the knowledge, attitudes, and practices of college students with regard to sun exposure hazards. The study showed that the majority of participants knew that sun exposure increased the risk for skin cancer. However, only 29% of participants correctly identified behaviors that reduce this risk (Spradlin et al., 2010).

In a separate study by Saridi et al. (2016) of 387 students at a university in Greece, the majority of students (91.7%) reported awareness of the risks of sun radiation exposure. Moreover, the vast majority of students (98.7%) knew that the risks involving serious damage increased due to sun exposure without protection. The main consequences of sun radiation identified by the students were skin cancer (97.7%), allergies (30.5%), skin aging (72.9%), and blinding (12.9%; Saridi et al., 2016).

Among our population study, there was a fairly satisfactory level of knowledge of sunscreen products as a protection measure, and 70.9% of students agreed that sunscreen products are not harmful to the vitamin D levels in their body. On the contrary, a misconception of the relationship between sunscreen and vitamin D production was displayed in a study conducted in Kuwait by Al-Mutairi et al. (2012). In addition, 73% of our students believed that a face cover (hijab) is not sufficient to replace sunscreen products. However, our study displayed a low level of knowledge with regard to the SPF and PA values of sunscreen products.

Sun tans were not prevalent among the students in our sample, and the majority (82.6%) had never experienced a sun tan. This is in line with a previous study conducted in Saudi Arabia by Allayali et al. (2014). In our population, a sun tan was not considered a sign of beauty. The majority did not believe that a sun tan increases levels of attractiveness. In fact, this study displayed a high level of awareness with regard to the harmful consequences of tanning beds, where 86.8% of students reported awareness about their harmful consequences. This result gives our group the advantage of lower chances of sustaining the side effects of sun tans.

Other studies provide contrary results, and show that students believe a sun tan makes them look more attractive, and thus intentionally expose themselves to the sun for the purpose of tanning. This disparity between results is due to differences in cultural background, as well as different beauty standards (Hobbs et al., 2014, Saridi et al., 2016, Urasaki et al., 2016). Despite the relatively high levels of knowledge among students and their healthy beliefs with regard to sun tans, the rate of sunscreen product use is low. Our results indicate that only 23.6% of students were using sunscreen product regularly.

According to experts, sunscreen products are considered the first line of defense against the harmful effects of radiation (Urasaki et al., 2016). The use of sunscreen products requires greater attention. Even those who actively engage in sun protection methods fall prey to misconceptions with regard to the correct usage of sunscreen products. For example, approximately 63% of students never reapply sunscreen products, and only 3% admit to regularly reapplying. A high percentage of students (64.9%) had never heard of SPF values. Moreover, 30.2% of sunscreen product users did not know the SPF value of their products. Other studies conducted in the United States (Hobbs et al., 2014), Brazil (Urasaki et al., 2016), and Greece (Saridi et al., 2016) had similar results.

These results emphasize the need for future campaigns to focus on the significant importance of sunscreen products, as well as the correct methods of application. To reach larger percentages of the population, we could exploit the media, particularly social media, and its enormous effects and influences to convey sun protection education messages.

To construct healthy prevention habits among society members, the effects of the sun and protective measures should be introduced in primary schools. Schools already possess the infrastructure and curriculum in which we can embed a new health education. In addition, individuals are more likely to adapt behavior more rapidly during childhood, because they can engage in activities that facilitate learning and develop lifelong habits to protect their skin from sun exposure.

When analyzing factors that deter individuals from using sunscreen products, the main reason was the uncomfortable feeling of the product on the skin. This feeling can be minimized by using suitable forms of sunscreen. Sunscreen can be found in the form of hydroalcoholic lotions, oils, oily gels, oil-in-water emulsions, water-in-oil emulsions, and sunscreen sticks and sprays, among others (Balogh et al., 2011).

Our study focused on women. However, men’s knowledge and practices in Saudi Arabia toward sun protection, as reported in previous studies conducted in Saudi Arabia, are lower than that of women, and lower with regard to sunscreen product use (Al Ghamdi et al., 2016, Al Robaee, 2010). This could be explained by the fact that most men think that skin care and sunscreen products are feminine. This result was in line with that of other studies conducted in different countries (Mousavi et al., 2011, Uğrlu et al., 2016).

## Limitations

This study was conducted with nonmedical female students at KAU, and can therefore not be considered as representative of the Saudi population. Furthermore, female students could be wearing makeup with SPF protection, which we did not include in our survey. Further research should include a larger sample taken from different geographic areas, and consider both sexes, as well as all ages and levels of education. However, lack of finances and human resources in our team limited our research to this sample.

## Conclusions

This study showed a good level of awareness among female university students on the harmful consequences of unprotected sun exposure, yet sun protection behavior and compliance to use sunscreen products is low. Seeking shade and wearing clothes were the methods most used to protect from the sun. The most commonly reported reason that deterred from using sunscreen was the discomfort of feeling of sunscreen products on the skin. The use of sunscreen products requires greater attention. Even those who actively engage in the use sun protection methods fall prey to misconceptions with regard to the correct use of sunscreen products.

These results emphasize the need for future campaigns that focus on the significant importance of sunscreen products, as well as the correct methods to apply the products.
